# The Effects of Health Information Technology on Quality of Care in Emergency Departments: A Systematic Review

**DOI:** 10.1002/hsr2.70962

**Published:** 2025-07-07

**Authors:** Clemens Scott Kruse, Michael Mileski, Brooke Herzog, Leah M. Frye, Jackson R. Spencer, Elizabeth A. Stevenson

**Affiliations:** ^1^ The University of Texas, El Paso Texas USA; ^2^ Texas State University, School of Health Administration, San Marcos Texas USA

**Keywords:** electronic health records, emergency departments, healthcare delivery, healthcare information technology, patient data, quality of care

## Abstract

**Background and Aims:**

To assess the effectiveness on quality of care when leveraging health information technology in the emergency department. Using MeSH terms, secondary data sources from CINAHL, PubMed (MEDLINE), Web of Science, and ScienceDirect were queried for the use of health information technology (HIT) in the emergency department and any effects on quality of care.

**Methods:**

This systematic review was conducted between June 2020 and January 2021 using the Kruse Protocol. It was reported using the Preferred Reporting items for Meta‐analyses and Systematic Reviews (PRISMA). Articles were considered eligible for analysis if they were published between January 1, 2016, and January 29, 2021, written in the English language, and if they reported effects of HIT usage on quality in the emergency department setting. No other intervention was utilized. A standardized Excel spreadsheet was used to extract data from 27 articles. A narrative analysis identified themes, which were reported in tables and summarized in affinity matrices.

**Results:**

Twenty‐seven articles were chosen to review. HIT tools, such as standardized procedures, protocols, checklists, health information exchange, and e‐prescribing, were introduced into the ED environment. These resulted in an increase in communication, clinical documentation, improved clinical handoffs, better prediction of falls and readmission, and a decrease in error.

**Conclusion:**

HIT implementation in emergency departments can improve workflow, documentation, individualized care, and communication between providers. EHRs can also reduce medical errors at the time of patient care. Quality training for providers in an EHR system may improve overall satisfaction with the implementation process.

## Summary

- What is known about this topic?
  ◦ Health information technology is expensive and its unwarranted use in the emergency department is disruptive.
  ◦ Although ancillary studies have shown moderate increases in quality because of HIT, training and EHR integration remain problematic.
- What this study adds?
  ◦ This study identifies additional benefits of communication in handoffs (between facilities and between ICU).
  ◦ This study identifies additional benefits from integrating standardized checklists and decision tools into the ED can introduce.

## 1 Introduction

### 1.1 Background and Rationale

The proliferation of technology in the medical care setting has progressed since the emergence of the mainframe, but successful implementation of information technology (IT) at the point of care is not consistent [1, 2]. Although health IT has been associated with positive medical outcomes for patients and improvements in population health, physicians do not always warm to its implementation [3, 4]. Electronic health records (EHRs) and other decision tools are expensive to purchase and maintain, they are complex in their implementation, they do not easily merge into physician workflows, and they often require greater amounts of administrative time to enter and finalize notes than their paper counterparts [5]. One particular area where HIT struggles is the emergency department (ED) [6]. Research supports higher quality care when the EHR is utilized in the ED, but it also shows that the ED is a difficult area in which to implement the EHR [6, 7, 8, 9]. The complexity and time‐sensitive nature of the ED introduces complication in the IT implementation process.

### 1.2 Significance

Use of HIT in the ED can account for efficiencies, cost savings, and rapid transfers to necessary care such as CCU/ICU. Leveraging HIT has the potential to increase patient‐centric care in a timely manner.

A systematic review was conducted in 2019 to illustrate the scope and influence of the EHR‐integrated clinical decision support system (CDSS) in the emergency department (ED). It reported positive outcomes: The most common were process measures directly related to workflows and rates of compliance with guidelines for imaging and medication orders [10].

Another systematic review was conducted in 2020. It examined health outcomes and healthcare efficiencies associated with the use of the EHR in EDs. It reported three types of efficiencies: clinicians saved time, reduction in unnecessary testing, and cost savings associated with the reduction in duplicate testing [11].

An observational cohort study was completed in 2020. It evaluated pediatric sepsis definitions designed for EHR extraction and multicenter quality improvement. It found that the complex nature of pediatric care resulted in immediate transfer to ICU beds in 60.8% of cases. Automatic EHR extraction accounted for critical data gathering and enabled such rapid transfers in 56.5% of participating hospitals. The remainder used manual charting, and the time to transfer was longer.

These studies illustrate the importance of leveraging the EHR in the ED, but these studies focus heavily on the EHR instead of the broader technology of HIT, and none of the studies collected data on medical outcomes, quality indicators, patient satisfaction, or barriers to adoption.

### 1.3 Study Objectives

The purpose of this systematic review was to examine the effect on quality that HIT creates in the ED for all patients compared with EDs that do not use HIT over the last 5 years. Quality was measured by patient satisfaction, length of stay, efficiency of care, improved communication, and fewer errors. Additionally, observations of patient satisfaction and barriers to adoption were explored.

## 2 Methods

### 2.1 Eligibility Criteria

Authors considered articles eligible for selection for analysis if they met the following conditions: (1) They were published between January 1, 2016 and January 29, 2021, (2) They were published in peer‐reviewed journals, (3) They were written in the English language, (4) They reported the effect of EHR usage on quality in the emergency department setting. Authors chose to define high‐quality healthcare as care that is safe, patient‐centered, timely, delivered by professionals, equitable, efficient, and increase the likelihood of desired outcomes [12]. Authors chose the publication period of 5 years due to the high number of articles available from our searches: any period longer than 5 years created a data set too large for our team to analyze and synthesize. Additionally, authors assessed quality of articles using the Johns Hopkins Nursing Evidence Based Practice rating scale (JHNEBP) [13]. Low‐quality studies were not eligible for analysis.

### 2.2 Information Sources

This review queried four common research databases: Web of Science, PubMed (Medline), the Cumulative Index of Nursing and Allied Health Literature (CINAHL), and Science Direct. Authors also queried the trial registry Clinicaltrials.gov to ensure the search did not miss any relevant studies.

### 2.3 Search

To write the introduction portion of this manuscript, reviewers queried Google Scholar. This was done to help define the problem and identify key terms for use in research databases. Using the Medical Subject Headings (MeSH) in PubMed, reviewers experimented with the key terms gathered from the Google Scholar search to find a suitable Boolean search string that would conduct an exhaustive search. The initial search for literature was conducted on June 10, 2020, and the final search was on January 30, 2021, using CINAHL, MEDLINE databases. The final Boolean search string was, (((“Electronic Health Records”[Mesh]) AND “Quality of Health Care”[Mesh]) AND “Emergency Medical Services”[Mesh]) AND “Emergency Department”[Mesh]. These terms were optimized to create an exhaustive search using relevant terms.

### 2.4 Study Selection

The Kruse Protocol uses three consensus meetings to discuss process and findings [14]. The first meeting finalized the group of studies to analyze. This was performed in an iterative manner. Authors downloaded results from the four database searches into a standardized Excel spreadsheet, and they used this tool for abstract screening. The authors asked the group leader to assign workload on the standardized spreadsheet to ensure each abstract was screened by at least two reviewers. Authors independently recorded recommendations on each article to keep it for analysis or discard it. For the latter, each author identified a code to identify the reason for discarding it. In case of a tie, the group leader chose another team member to read the article and make a recommendation. The consensus meeting discussed disagreement and made final determinations of articles for analysis.

### 2.5 Data Collection Process and Data Items

Following the Kruse Protocol, the second and third consensus meetings were organized [14]. The group leader assigned workload on the final group of articles to ensure each article was analyzed by at least two reviewers. Using the standardized Excel spreadsheet as the data extraction tool, the following fields of data were collected: (PICOS) Participants used, Intervention employed, Comparison (to control) found, Outcome of study, Study design used, country of origin, statistics used for analysis, the JHNEBP quality assessment, observations of bias, and general observations in the areas of quality and medical outcomes used to identify themes. Authors used the second consensus meeting to discuss observations. They conducted a narrative analysis to make sense of the observations, and they identified themes [15]. Reviewers read through their articles another time using the common set of themes to watch for. During the third consensus meeting, themes were discussed and compared to identify interactions and make inferences. Cochran's *Q* was checked for all studies to ensure the results reported were above the rejection criteria. Because Zotero was used to track references, it checked to ensure all articles selected had not been retracted.

### 2.6 Risk of Bias Within and Across Studies

In addition to making general observations of bias, authors used the JHNEBP tool to assess the level of quality of the articles analyzed [13]. Authors assumed that inferences from lower quality articles posed a threat to external validity, so they were discarded. In general, the JHNEBP tool categorizes the strength of evidence of articles across five levels and quality of evidence across three levels. Levels 1 and 2 were reserved for randomized control trials (RCTs) and quasi‐experimental studies. Level 3 identified nonexperimental, qualitative, or meta‐synthesis studies. Levels 4 and 5 identified opinions based on nationally recognized experts: specifically, four was opinions based on research evidence or consensus panels, and five was opinions not based on research. The three quality scales (A–C) ranged from high to low. They are distinguished from the others in the areas of research, summative reviews, organizational, and expert opinion. To qualify for Level A, a study must demonstrate consistent results with adequate control, sufficient sample size, and conclusions that are definitive. Research assessed at level C, however, presents little evidence, and it often comes with an insufficient sample size, inconsistent results, and conclusions that cannot easily be drawn from the data. Articles assessed at level 4 were not eligible for analysis. Articles assessed at level C were discussed for a decision on inclusion.

### 2.7 Summary Measures

Authors considered articles eligible for analysis if they were quantitative, qualitative, or mixed methods. This meant that summary measures were not consistent across the group of articles for analysis. Authors preferred that the summary statistic would be the risk ratio, but they also accepted descriptive statistics and means comparisons (e.g., student's‐*t*).

### 2.8 Additional Analysis

The narrative analysis conducted in the second consensus meeting drove the additional analysis. After reviewers analyzed articles another time, themes were more thoroughly flushed out of the literature. Themes were organized into affinity matrices reporting summary statistics.

## 3 Results

### 3.1 Study Selection

**Figure 1:**
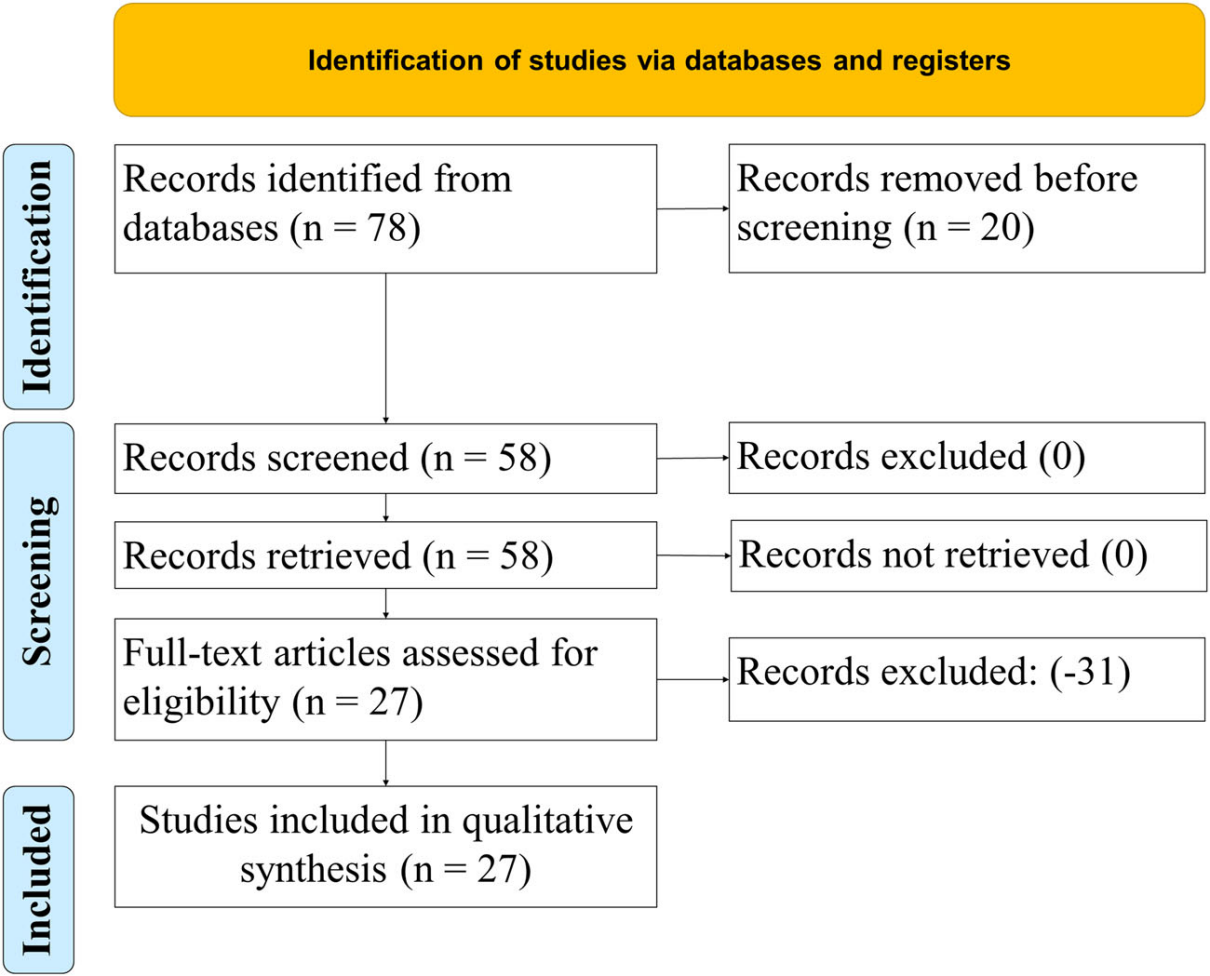
PRISMA flow diagram showing article selection process.

Figure 1 outlines the article selection process. The initial search resulted in 215 abstracts that were divided and screened among the reviewers twice. During abstract review, objectives were addressed, and notes were taken on a Microsoft Excel spreadsheet. The notes on the Excel spreadsheet were combined to reflect agreement or disagreement on whether the articles were considered germane. Authors calculated a *k* statistic to reflect a level of agreement. This was calculated after the first consensus meeting (*k* = 0.95). This kappa level illustrates near perfect agreement [16, 17]. At the end of the first consensus meeting, 27 articles were chosen for analysis.

### 3.2 Study Characteristics

Using the standardized Excel spreadsheet, authors extracted data items for PICOS: participants, intervention, comparison, outcome, and study design (see Table 1). Authors also collected observations of quality of care, patient satisfaction, barriers to adoption, and medical outcomes associated with the use of HIT in the ED. These items are reported in chronological order: 2016 [18, 19, 20, 21, 22, 23, 24, 25, 26], 2017 [27, 28, 29, 30, 31], 2018 [32, 33], 2019 [34, 35, 36, 37, 38, 39, 40], 2020 [41, 42, 43, 44]. Articles assessed for eligibility from 2021 were deemed not sufficiently germane to the topic.

**Table 1:** PICOS – Characteristics of each study.

Authors	Participants	Intervention	Intervention themes	Results (Compared to control group)	Medical outcomes reported	Study design
Balhous, et al. [18]	170 children (6 months to 18 years) referred to a pediatric ED for childhood diseases.	The Health information Exchange (HIE) system; The referral letters; The patients' medical history were analyzed to assess information gaps	Health information exchange	No control group. The following were indicators of key areas: The HIE system (*x̄* = 54.1%); The referral letter (*x̄* = 43.9%); The patient's medical history (*x̄ *= 73.5%).	The HIE, referral letters, and medical history can narrow information gaps and may reduce the time required for collecting patient's medical information at time‐critical points	Retrospective cohort
Byczkowski, et al. [19]	68 Adults (parents, 20–40 + ), accompanying their child to the ED. Large tertiary‐care pediatric health system. Purposive sampling used.	None. Focus group identified through the EHR, and described through a survey dimension of family‐centered care important to parents in pediatric emergency care	Checklist	Researchers compared their results to published study populations. Family‐centered care divided into eight dimensions: (1) emotional support, (2) coordination of care, (3) involve the family and patient in care decisions by prompting for and respecting care preferences, (4) alternative and timely care, (5) education, communication, and information, (6) management of pain, (7) child‐focused and safe care, (8) continuity	Not reported.	Qualitative Delphi
Chan, et al. [20]	3065 adults who were 72‐h readmissions.	None. Decision tool developed using the EHR.	Decision tool	The control group was non readmissions compared based on patient demographics, mode of arrival, acuity of patient, status of category, seniority ranking of physician in charge, and medical diagnosis. Risk factors identified with returning within 72 h.: older males who arrive by ambulance and triaged as P2, and diagnoses of heart problems, abdominal pain, or viral infection (*p* < 0.001); Chinese ethnicity (*p* = 0.006). Seniority of physicians showed no significant differences between groups (*p* = 0.419).	Not reported.	Retrospective cohort
Horner, et al. [21]	21,152 adolescents with abdominal pain presented for care in the ED and sent for images	None. Checklist developed using EHR to predict the likelihood of imaging.	Checklist	No control group. Imaging was performed 29.7% of the visits; Isolated CT 13.4% of visits. There were differences by several factors: The Black (OR: 0.4 (95% CI: 0.4, 0.5)), Medicaid (OR: 0.6 (95% CI: 0.5, 0.7)) patients showed lower odds of advanced imaging when compared to white patients with private insurance, respectively.	Racial and insurance disparities were present. General EDs are less likely than pediatric EDs to use advanced imaging overall.	Retrospective cohort
Khalil, et al. [22]	56 adults with the following characteristics: Admitted to an Acute Assessment and Admission Unit; Via the ED; an environment that uses electronic medication management; and metropolitan Australian hospital.	The electronic prescribing and medication administration (MAR) reconciliation.	Electronic prescribing	Control group (*n* = 54). Decreased average error rate from 4·41 to 0·52 err/patient (*p* < 0.0001) and 0.43‐.05 errors per order (*p* < 0.005).	Reduction in the severity of the errors.	Prospective, observational
Manias, et al. [23]	127 adults (rural transfers). transcribed telephone handovers (‘patient expect’ calls) occurring with interhospital transfers from two rural hospitals to an Australian metropolitan tertiary hospital	Transcribed telephone handoffs followed by EHR transcription.	Standardized procedure	No control group. Patient conditions do not deteriorate as quicky when effective telephone handoffs occur during in‐flight transfers followed by EHR transcription.	Deterioration of condition was reported in three calls	Retrospective, observational
Matthaeus‐Kraemer, et al. [24]	29 adult clinicians participated in five multicenter focus group discussions	None. Standardized procedure developed for sepsis guidelines implementation (EHR provided records for group discussions)	Standardized procedure	No control group. The root cause of delayed detection was communication errors and handover difficulties throughout	Improved medical outcomes are associated with early recognition of sepsis followed by prompt therapy. Length of stay or mortality benefits not associated with 24/7 intensivist staffing	Qualitative Delphi
Melvin, et al. [25]	51 adult ED physicians who were users of CeHa‐HIE; 11 EDs were operated by four hospital systems: One academic ED, Five community hospital EDs, Four freestanding EDs, and one ED/chest pain center.	None. Survey instrument evaluated usability perceptions of the CeHa‐HIE.	Health information exchange	No control group. Most agreed the HIE was easy to use, and its use added value to their work in the ED. Physicians asked for improved information integration into system EHR.	Not reported.	Qualitative Delphi
Tsai, et al. [26]	51 adult ED patients ≥ 18 years or older with ED revisit within 72 h, patients also experienced a subsequent admission to the ICU.	None. Decision tool developed with the EHR.	Decision tool	No control group.	Most patients visited the ED in the evening (51%); Identified as Triage Level III (76.5%) in their first ED visit. Causes of revisits followed by ICU admission were doctor‐related (21/51, 41.1%), illness‐related (18/51, 35.3%), or patient‐related (12/51, 23.5%). Disease categories: neurological (23.5%), digestive (23.5%), and cardiovascular (21.6%).	Retrospective observational
Everson, et al. [27]	2163 adult ED patients at a large academic medical center (Clinicians used information from outside their EHR)	HIE. Researchers used the audit data from an EHR to capture the time span between the request for outside information and access of the data. Researchers assessed whether length of stay, odds of imaging, hospitalization, and total charge was mediated by the measured request‐to‐access time.	Health information exchange	No control group. Researchers found no direct association between the mode of return of HIE information and ED outcomes. Use of the HIE was associated with faster request‐to‐access (*x̄* = 58.5 min); and faster access resulted in faster ED service; Each 1 h shorter access to information resulted in shorter ED stay (*x̄ *= 52.9 min), likelihood of imaging (*x̄* = 2.5%, 1.6%, and 2.4% for CT, MRI, and radiographs, respectively), and likelihood of admission was 2.4% lower, (*p* < 0.001).	Use of the HIE reduced time‐to‐access, which improved both care processes and ED utilization.	Retrospective observational
Harris, et al. [28]	129 Older adults in 260 ED episodes of care, > 60, at a metropolitan, 300 bed district hospital in an outer suburb with a 22‐bed referral center ED.	Checklist for Parkinson's Disease and falls associated with other patients in ED. (EHR provided data)	Checklist	No control group. No statistically significant difference in triage code or representation rate between patients. Time to be seen *x̅ *= 23 min (SD 26); Time to be seen between business hours and after hours was *x̅* = 20 (SD 21) and *x̅* = 30 (SD 32), respectively. Patients under 80 yrs (*n* = 132; 51.0%) were admitted to hospital more frequently than those over 80 yrs (Pearson's *X* ^2^ test 162.2; df 1; *p* = 0.001).	Documentation of medication regimes, adverse outcomes, and emergency care are improved when providers recognize a PD presentation.	Retrospective observational
Josephy, et al. [29]	381 Adults sedated in ED	Standardized procedure of single vs. two physician sedation (EHR provided the pre data in the pre‐post design)	Standardized procedure	Single physician sedation resulted in zero adverse events when compared with two physician team.	Single physician sedation resulted in zero adverse events	Retrospective (pre) and prospective (post) pre‐post single‐center observational
Newcomb, et al. [30]	304 Adult frequent users of the ED at an urban medical center compared with 304 infrequent users.	Prescribing protocol developed. User responses were compared on patient satisfaction surveys, specifically for pain complaints. (EHR provided patient matches)	Standardized procedure	No control group. The frequent ED users were 75% less likely to return a satisfaction survey than the infrequent ED users (OR = 0.2488; *p* < 0.0001).	ED providers can feel confident that they can withhold opioids without concern of adversely affecting patient satisfaction.	Retrospective cohort
Okafor, et al. [31]	164 care transitions of adults with the following conditions: urban tertiary care, level 1 trauma center, 60,000 ED visits annual, EM residency program.	Standardized care transition process and EMR monitoring.	Standardized procedure	Pre intervention revealed 94 relevant missed clinical terms (MCI) while post intervention showed only 36 MCI with a similar number of care transitions. This was a 58% decrease in MCI without an increase in time for transitions. Users of the standardized procedure reported high satisfaction with care transitions.	The primary outcome measure was the number of missed clinical items. Both residents and faculty felt the intervention decreased the number of MCI.	Prospective pre‐post
Bennet, et al. [32]	5419 adult patients, 2933 with rare conditions (Spina Bifida, Muscular Dystrophy, and Fragile X Syndrome) and 2486 without rare conditions who presented for care at the ED. The 30‐day readmission rates were compared	Decision tool. The EHR provided the data.	Decision tool	Through logistic regression, authors showed a statistically significant difference in 30‐day readmission rates between groups. Rare conditions contributed significantly.	Rare conditions resulted in more inpatient visits.	Case control analysis. Quasi‐experimental
Brice, et al. [33]	160 short‐stay hospitals, ED visits of Medicare beneficiaries.	Meaningful Use of technology (MU).	Meaningful Use	The average EHR adoption among physicians was 43%. Post‐acute utilization indicators improved.	30‐day readmissions and 30‐day ED utilization was indistinguishable between hospitals with MU and those without MU of technology.	Interrupted time‐series, Quasi‐experimental
Brauer, et al. [34]	440,742 Adults discharged after of the rectum, colon, or small intestine operations. The two primary outcomes were death and 30‐day readmission.	LACE model. The EHR provided data to the State Inpatient Database of the Agency for Healthcare Research and Quality (AHRQ), and Healthcare Cost and Utilization Project (HCUP) for Florida, California, and New York for the years 2006‐2014, 2006‐2011, and 2006‐2013, respectively.	Standardized procedure	The rate of death or 30‐day readmission was 14% (*p* < 0.494). The LACE model was a poor fit between groups (*C* = 0.631), and adding additional variables only slightly improved it.	The rate of death or readmission within 30 days after discharge was not different between the groups (*C* = 0.631).	Retrospective split sample cohort
Bui, et al. [35]	1342 CMS beneficiary adults with at least one chronic condition (not pregnant) in Baltimore. Primary outcomes were ED, hospitalizations, and 30‐day readmissions.	Standardized procedure (Barriers to Care). Two intervention exposures were compared (# contacts with case manager or community health worker: ≤ 1/month or > 1/month).	Standardized procedure	There was higher systolic blood pressure in the high contact group. Medicaid participants in the high contact group had 42% (rate ratio (RR): 1.42; 95% CI: 1.08–1.86) and 64% (RR: 1.64; 95% CI: 1.08–2.48) greater risks for hospitalization and readmission than the low contact group, respectively.	Medicaid participants in the high contact group had 42% (rate ratio (RR): 1.42; 95% CI: 1.08–1.86) and 64% (RR: 1.64; 95% CI: 1.08–2.48) greater risks for hospitalization and readmission than the low contact group, respectively.	Quasi‐experimental
Curtis, et al. [36]	63 Adult ED nurses plus senior medical officers working at three EDs in Australia.	History, Identify Red flags, Assessment, Interventions, Diagnostics, Reassessment, Communication (HIRAID) evaluated with ED nurses.	Decision tool	The self‐efficacy score did not change between groups (*x̅* (SD): 8.79 (1.12) vs. 9.03 (0.85), *t* = 0.91, *p* = 0.365).	Not reported.	pre‐post
Economos, et al. [37]	146 adults given end‐of‐life care in ED at a French primary care hospital	Standardized procedure. The EHR provided data. Participants were categorized by actively dying or not‐actively dying, based on provider comments.	Standardized procedure	There was no statistical difference between end‐of‐life care for actively and non‐actively dying patients.	Actively dying patients mostly suffered from vascular conditions (29.4%), were more likely to have decisions to withhold or withdraw treatments (OR = 5.3 [1.56; 20.7], *p* = 0.003), to have strong opioids (OR = 5.32 [2.1; 13.9], *p* <0.001), hypnotics (OR = 2.6 [0.95; 8.39], *p* = 0.05), scopolamine (OR = 2.5 [1.1; 6.13], *p* = 0.03), were less likely to have unbeneficial treatments in terminal conditions, such as resuscitation care (OR = 0.06 [0.001; 0.52], *p* = 0.002) and antibiotics (OR = 0.42 [0.19; 0.92], *p* = 0.022).	Retrospective cohort
Martinez‐Sanchez, et al. [38]	254 adolescents ≤ 18 treated by an advanced life support (ALS) in the ED for poison exposure and treated in Catalonia.	Quality decision tool. Researchers evaluated eight quality indicators (QI) for care received prehospital ED. Data collected from the EHR.	Decision tool	No control group. Intervention teams: (EMT + ALSn, technician + ALSn, or technician + physician) Observations were CO2 (33.8%), intentional poisoning (124, 50.8%), and serious poisoning symptoms (42, 16.5%). Treatments applied: O2 therapy (82, 32.3%), fluid therapy (46, 18.1%) and administration of antidotes (17, 6.7%).	Decreased time to treatment.	Prospective Observational
Olino, et al. [39]	8028 adults in a Brazilian teaching hospital	Standardized procedure. Transfer Notes (NT) in the EHR along with Modified Early Warning Scores (MEWS) analyzed for effective communication.	Standardized procedure	No control group. The NT was performed at 95% in only 2 months (Jan–Feb). The MEWS was recorded in 85.6% (*n* = 6870) of the EHR records. The MEWS remained unchanged in 96.8% (*n* = 6652) EHR records.	Not reported.	Retrospective cross sectional
Yakusheva, et al. [40]	29,986 Adult patients and 522 nurses at 31 geographically diverse medical‐surgical units.	Decision tool from the READI (Readiness Evaluation and Discharge Interventions) study. Patient discharge data from the EHR provided eight item short form of READI. The 30‐day readmission or ED visit was used as a categorical variable.	Decision tool	No control group. There was a negative association between nurse productivity and the likelihood of a readmission (−0.48 percentage points, *p* < 0.001) and an ED visit ( − 0.29 percentage points, *p* = 0.042). The variability in nurse productivity explained only 9.07% of the variance in patient discharge readiness scores.	Not reported.	Retrospective observational
Delawder, et al. [41]	214 adults presenting to the ED with sepsis, severe sepsis, or septic shock.	QI Checklist ‐‐ sepsis prevention built into EHR. Develop and implement an interdisciplinary team to address early implementation of sepsis bundles in the emergency department and to compare sepsis bundle compliance 3 months pre‐ and 3 months postintervention implementation.	Checklist	The intervention showed an improvement in time to each bundle element, except for antibiotics and blood cultures. Changes were also observed in meeting bundle compliance in fluid resuscitation volume (*χ* ^2^ = 16.3, *p* ≤ 0.001): initial lactate collected within 180 min (*χ* ^2^ = 11.3,*p* ≤ 0.01) and time to second lactate within 360 min (*χ* ^2^ = 27.7, *p* ≤ 0.001).	Mortality rates declined from 12% to 5%.	Rapid‐cycle quality improvement, pre‐post
Dimeff, et al. [42]	24 adult suicidal patients admitted to ER for evaluation.	VCAM decision tool integrated with EHR. The “Dr. Dave” avatar was an iterative, user‐centered design. This along with other patient‐facing tools, provider‐facing tools, and a clinical decision support (CDSS) tool were evaluated to aid discharge disposition.	Decision tool	No control group. Patients scored highly on the Perception of Care domain (12/18, 66.7%) endorsed this domain at a medium to high level. The inter‐rater reliability *k* = 0.900; *p* < 0.0001).	Not reported	nonexperimental (no randomization, no control). This was a proof‐of‐concept using iterative design.
Munjal, et al. [43]	207 Older adults ( ≥ 65, median 83) 65% female, ambulance transported home after discharge from hospital. The match group was 162 older adults NOT transported by EMS.	Standardized procedure. Measured outcomes: EMS transported home; 72 h. and 30‐day ED revisits and 30‐day readmissions. Data provided by the EHR.	Standardized procedure	A higher rate of 30‐day ED revisit observed in the intervention group (18.52% vs. 10.49%; OR 1.939; *p* = 0.043). A higher rate of 72‐h ED returns was also observed by EMS‐transported discharges (2.47% vs. 0.62%; OR 4.076; *p* = 0.21) and 30‐day readmissions (12.35% vs. 6.17%; OR 2.141; *p* = 0.06). This result was not statistically significant.	Discharges transported home by EMS are at a higher risk of 30‐day ED revisit, 72 h. ED return, and 30‐day readmission.	Retrospective cohort, quasi‐experimental
Wooldridge, et al. [44]	18 adult ED providers at a level 1 trauma center (111 beds, 8 pediatric operating rooms, a 21‐bed PICU) using 34 patient transfers.	Systems Engineering Initiative for Patient Safety (SEIPS) checklist. It was developed using semi‐structured interviews.	Checklist	The checklist development process identified nine dimensions of work system barriers and facilitators in care transitions of pediatric trauma patients from the ED to the operating room (13 cases), operating room to PICU (12 cases) and ED to PICU (9 cases): ED decision making, anticipation, role ambiguity, interacting with family, staffing/resources, physical environment, technology, team cognition, and characteristic of trauma care. At least one dimension was observed in all work‐system elements.	Not reported.	Qualitative, Delphi

### 3.3 Risk of Bias Within and Across Studies

Researchers assess quality of research and risk of bias through the Johns Hopkins Nursing Evidence Based Process (JHNEBP). This tool was not automated. It was a manual process of screening articles‐based study design, and strength of sample commensurate with conclusions. Bias was also identified through close examination of each study. 70% of the studies assessed fell into strength of evidence III category (nonexperimental, qualitative, or meta‐analysis). About 56% of the studies assessed fell into quality of evidence B category (Good), which means there reasonably consistent results and definitive conclusions were reached using a sufficiently large sample size, some means of control.

In addition to the JHNEBP, authors made note of bias identified within each study. The types of bias identified were: Selection bias (23/27, 85%), sample bias (3/27, 11%), and design bias (1/27, 4%). The dangers of selection and sample bias in research are in external validity. Researchers and practitioners must carefully examine their population along with the sample in the study to ensure each study's results can be appropriately applied to their population. Finally, design bias was observed in one study. This study had a design flaw because it used care report summaries from their hospital that incurred information loss in transfer from their EHR to their analysis system. The dangers this imposes on research are on both internal and external validity.

Cohen's *Q* was evaluated for each study to ensure all results were below the rejection criteria. Because none of the articles selected were of low quality, Cohen's *Q* was satisfactory, indicating no heterogeneity observed in the results of individual articles.

### 3.4 Results of Individual Studies

Table 2 provides a summary of the results of each study analyzed as it relates to the objective statement. The summary includes the narrative analysis and the identification of themes in the literature. A direct translation of observation to theme is listed in Appendix SA. This appendix depicts the identification of the theme based on the individual observations from the review team. Appendix SB provides a translation of observations of patient/staff satisfaction, quality outcomes, and barriers to adoption to themes. Appendix SC provides a table of other observations made during the review such as bias, effect size, country of origin, statistics used, strength of evidence, and source of funding. Appendix SD provides a list of articles rejected by the search and selection process.

**Table 2:** Summary of article themes.

Authors	Intervention themes	Results themes	Medical outcomes themes	Satisfaction themes	Quality themes	Barrier themes
Balhous, et al. [18]	Health information exchange	Decrease in time to treatment	Improve the process of care	Not reported	Increase in communication or documentation	Requires integration with EHR
Byczkowski, et al. [19]	Checklist	Improved communication or reporting	Not reported	Satisfied or highly satisfied	Not reported	Not reported
Chan, et al. [20]	Decision tool	No improvement in readmission rates	Not reported	Not reported	Decrease in ED utilization	Not reported
Horner, et al. [21]	Checklist	No improvement in readmission rates	Improve the process of care	Not reported	Increase in communication or documentation	Not reported
Khalil, et al. [22]	Electronic prescribing	Decrease in error	Decrease in error	Satisfied or highly satisfied	Decrease in error	Not reported
Manias, et al. [23]	Standardized procedure	Improved communication or reporting	Improve the process of care	Not reported	Increase in communication or documentation	Not reported
Matthaeus‐Kraemer, et al. [24]	Standardized procedure	Improved communication or reporting	Improve the process of care	Not reported	Increase in communication or documentation	
Melvin, et al. [25]	Health information exchange	Positive perception of care or tool	Not reported	Satisfied or highly satisfied	Ease of use	Requires integration with EHR
Tsai, et al. [26]	Decision tool	No difference in quality of care or readmission rates	No difference in readmission	Not reported	Not associated with readmission	Not reported
Everson, et al. [27]	Health information exchange	Decrease in time to treatment	Improve the process of care	Not reported	Efficiency	Requires integration with EHR
Harris, et al. [28]	Checklist	Decrease in time to treatment	Improve the process of care	Not reported	Increase in communication or documentation	Not reported
Josephy, et al. [29]	Standardized procedure	No difference in quality of care or readmission rates	Improve the process of care	Not reported	Increase in communication or documentation	Not reported
Newcomb, et al. [30]	Standardized procedure	Affected pts less likely to report satisfaction	Improve the process of care	Satisfied or highly satisfied	Increase in communication or documentation	Not reported
Okafor, et al. [31]	Standardized procedure	Improved communication or reporting	Improve the process of care	Positive perception of care	Increase in communication or documentation	Not reported
Bennet, et al. [32]	Decision tool	Decrease in readmission rates	Increase in readmission	Not reported	Decrease in readmission	Not reported
Brice, et al. [33]	Meaningful Use	No improvement in readmission rates	Not reported	Not reported	Not associated with readmission	Cost
Brauer, et al. [34]	Standardized procedure	No difference in quality of care or readmission rates	No difference in mortality	Not reported	No difference in mortality	Requires integration with EHR
Bui, et al. [35]	Standardized procedure	No improvement in readmission rates	Improve the process of care	Not reported	Increase in communication or documentation	Not reported
Curtis, et al. [36]	Decision tool	No difference in quality of care or readmission rates	Not reported	Satisfied or highly satisfied	Increase in communication or documentation	
Economos, et al. [37]	Standardized procedure	No improvement in readmission rates	Improve the process of care	Not reported	Decrease in error	
Martinez‐Sanchez, et al. [38]	Decision tool	Decrease in time to treatment	Improve the process of care	Not reported	Increase in communication or documentation	Cost
Olino, et al. [39]	Standardized procedure	Improved communication or reporting	Not reported	Not reported	Increase in communication or documentation	Requires integration with EHR
Yakusheva, et al. [40]	Decision tool	Negative productivity	Not reported	Not reported	Efficiency	Not reported
Delawder, et al. [41]	Checklist	Decrease in time to treatment	Decrease in mortality	Not reported	Decrease in mortality	Not reported
Dimeff, et al. [42]	Decision tool	Positive perception of care or tool	Not reported	Positive perception of care	Efficiency	Requires integration with EHR
Munjal, et al. [43]	Standardized procedure	No improvement in readmission rates	Increase in readmission	Not reported	Not associated with readmission	Cost
Wooldridge, et al. [44]	Checklist	Improved communication or reporting	Not reported	Not reported	Not reported	Affects ED decision making

### 3.5 Results of Synthesis

Only two studies reported an effect size, so a meta‐analysis was not possible.

### 3.6 Additional Analysis

#### 3.6.1 Interventions of HIT

**Figure 2:**
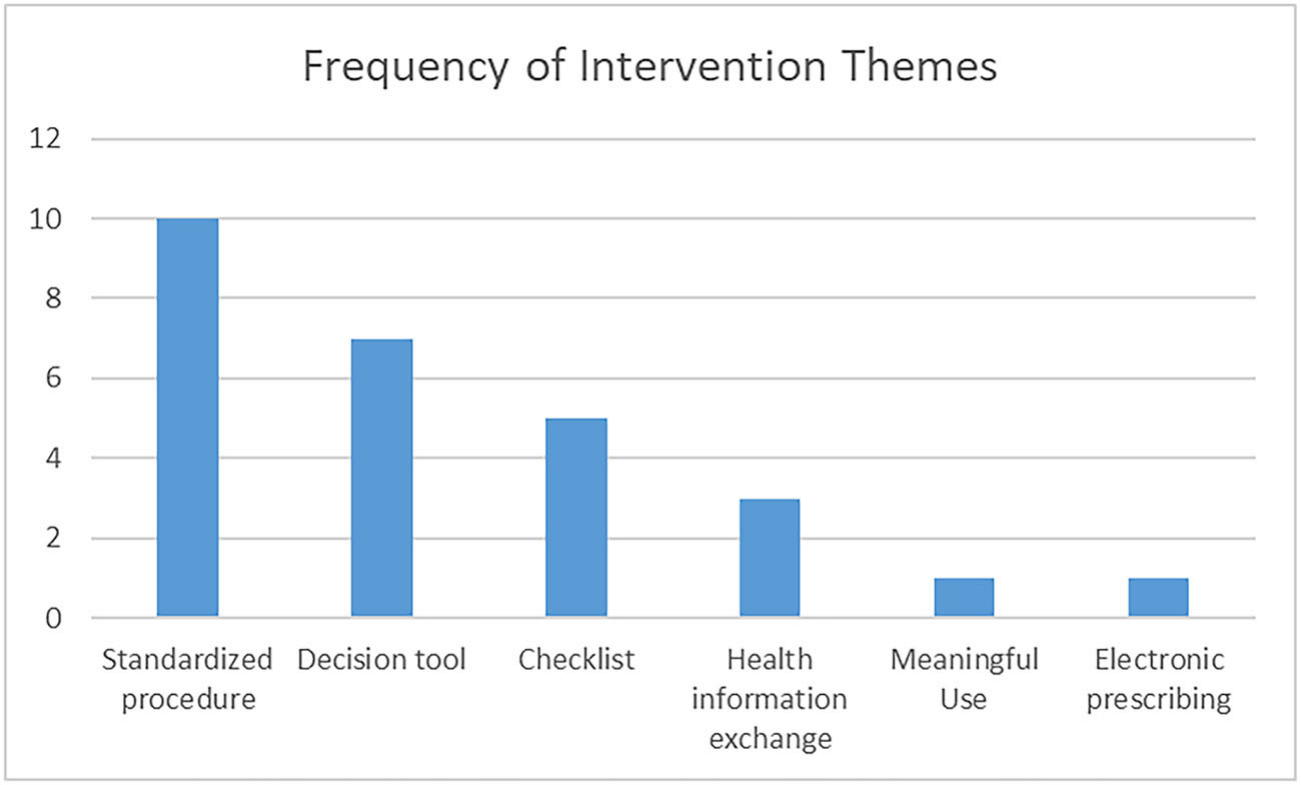
Chart of intervention themes identified in the literature.

Figure 2 charts the interventions identified in the literature.

Researchers identified six primary interventions (four themes and two observations): Standardized procedures (10/27, 37%) [23, 24, 29, 30, 31, 34, 35, 37, 39, 43], a decision tool for assessment of risk or discharge (7/27, 26%) [20, 26, 32, 36, 38, 40, 42], a checklist to assess risk of readmission (5/27, 19%) [19, 21, 28, 41, 44], the use of health information exchange (3/27, 11%) [18, 25, 27], meaningful use qualification [33] and electronic prescribing [22] each occurred 1/27, 4%. Of the six interventions identified, the standardized procedure showed the greatest level of benefit to quality of care. This came from an increase in communication, documentation, and improved clinical handoffs. The decision tools were often developed through Delphi studies, and these tools helped identify high‐risk factors for risk, discharge, and readmission. Integrating health information exchange and electronic prescribing at the ED enabled shorter treatment times and higher patient satisfaction.

### 3.7 Themes of Patient/Staff Satisfaction

Satisfaction of either patient or staff was rarely reported in the literature, but when reported, the result was positive. Patients and staff were either satisfied or highly satisfied with the intervention (5/27, 19%) [19, 22, 25, 30, 36], or there was a positive perception of care provided (2/27, 7%) [31, 42].

### 3.8 Theme of Quality

**Figure 3:**
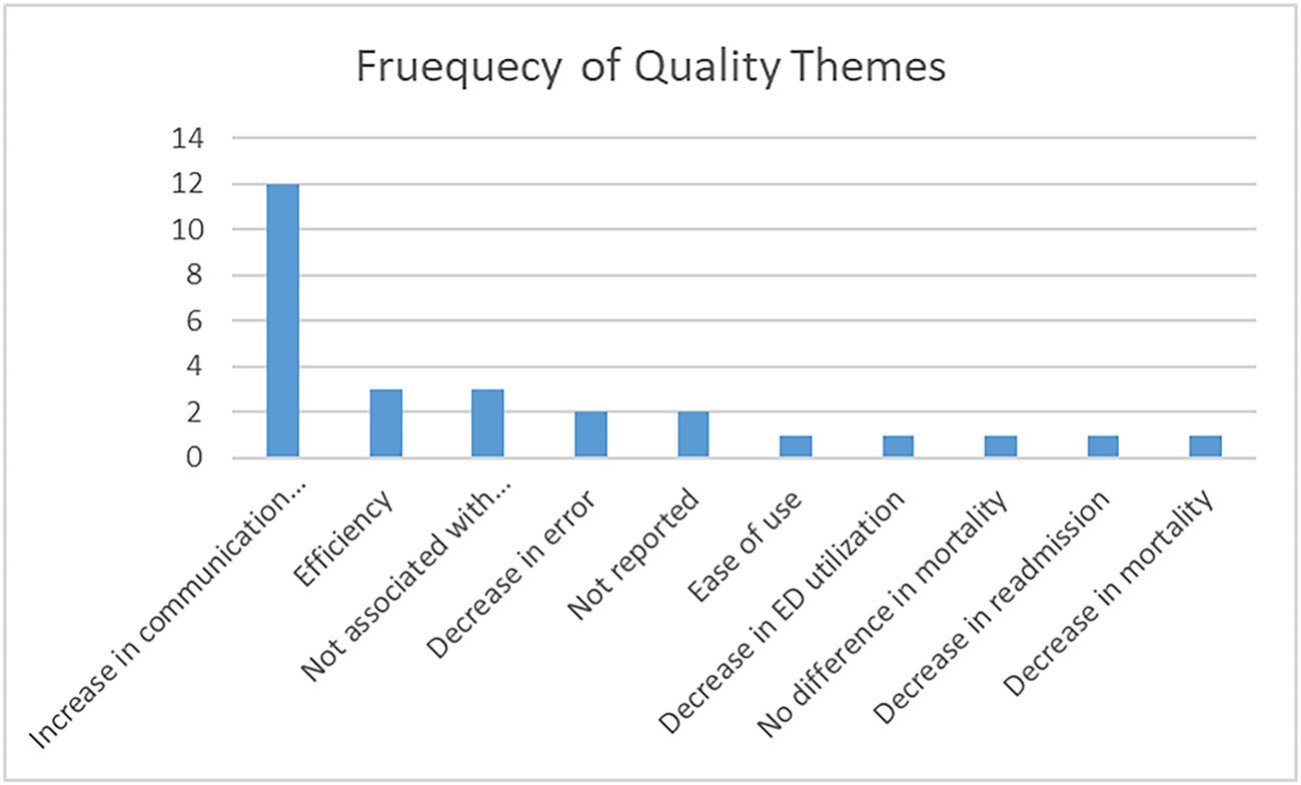
Chart of the frequencies associated with quality themes.

Four themes and five observations were recorded for quality outcomes. The most prevalent was the intervention increased communication or clinical documentation (12/27, 44%) [18, 21, 23, 24, 28, 29, 30, 31, 35, 36, 39]. The intervention introduced efficiencies of care [27, 40, 42], and the intervention was not associated with readmission each appeared in the same frequency (3/27, 11%) [26, 33, 43]. The intervention resulted in a decrease in error occurred 2/27, 7% [22, 37]. Several individual observations could not be associated with a theme: ease of use, decrease in ED utilization, decrease in readmission, decrease in mortality, and no difference in mortality [20, 25, 32, 41]. Table 3 tabulates the quality themes and Figure 3 charts the data.

**Table 3:** Summary of quality themes, sorted in order of frequency (not reported at the bottom).

Quality themes	Frequency
Increase in communication or documentation [18, 21, 23, 24, 28, 29, 30, 31, 35, 36, 39]	12
Efficiency [27, 40, 42]	3
Not associated with readmission [26, 33, 43]	3
Decrease in error [22, 37]	2
Ease of use [25]	1
Decrease in ED utilization [20]	1
No difference in mortality [34]	1
Decrease in readmission [32]	1
Decrease in mortality [41]	1
Not reported	2
	27

### 3.9 Themes of Barriers

**Figure 4:**
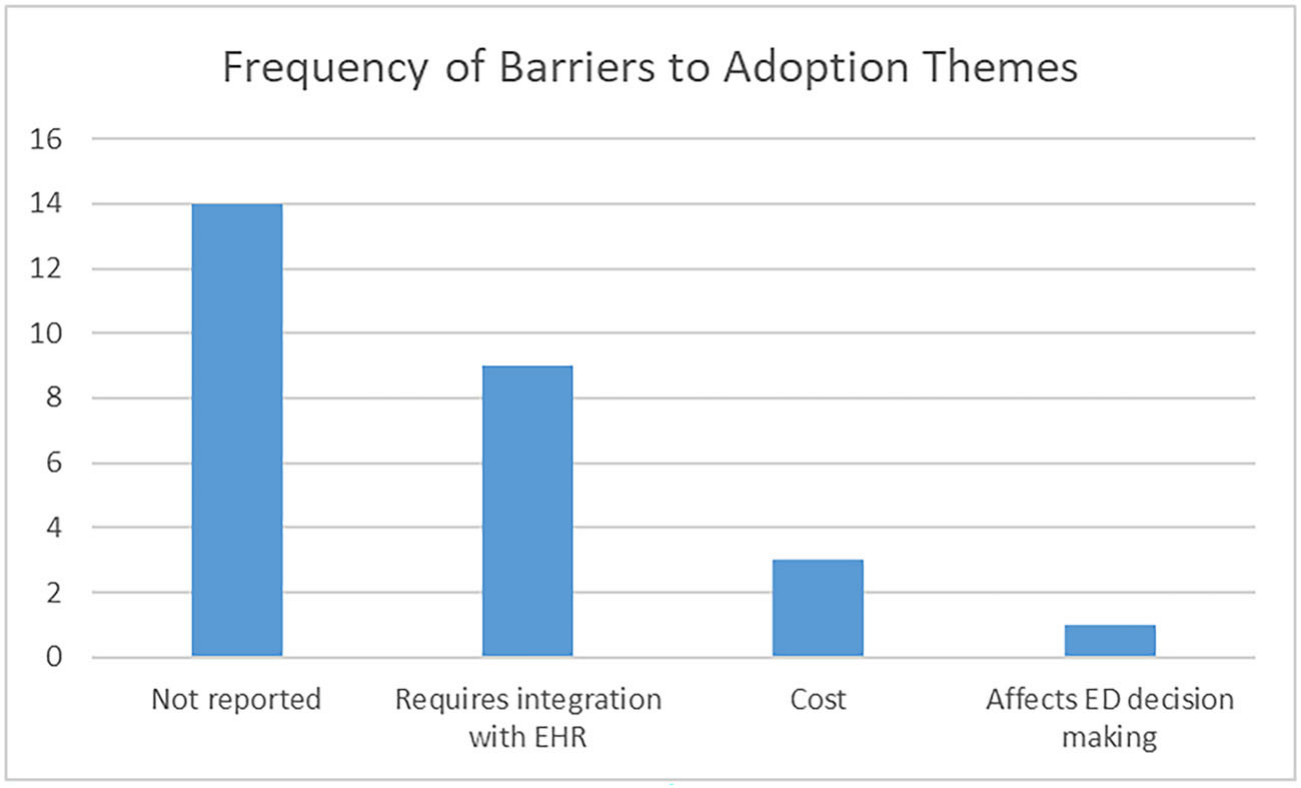
Frequency of themes for barriers to adoption.

Only two themes were identified for barriers to implementation: Requires integration with the EHR (9/27, 33%) [18, 24, 25, 27, 34, 36, 37, 39, 42] and cost (3/27, 13%) [33, 38, 43]. One observation could not be associated with a theme: The intervention affects ED decision making [44]. Figure 4 charts these frequencies.

### 3.10 Themes of Medical Outcomes

**Figure 5:**
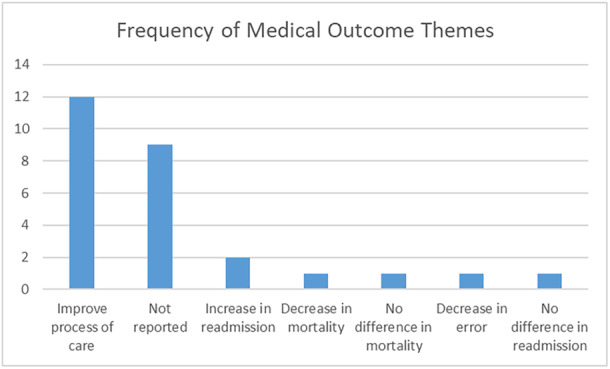
Frequencies of themes for medical outcomes.

Two themes and four observations were noted for medical outcomes. The most common theme was a general improvement in the process of care (12/27, 44%) [18, 21, 23, 24, 28, 29, 31, 35, 37, 38]. This often occurred due to the increase in clinical documentation inherent to the EHR and other integrated HIT decision tools. In two cases, the intervention tool helped predict an increase in readmission (2/27, 7%) [32, 43]. Other observations could not be associated with a theme: decrease in mortality, no difference in mortality, decrease in error, and no difference in readmission [26, 32, 34, 41, 43]. Table 4 tabulates the medical outcomes themes and Figure 5 charts them.

**Table 4:** Summary of medical outcomes associated with EHR adoption in the ED, sorted in order of frequency (not reported at the bottom).

Medical outcomes themes	Frequency
Improve process of care [18, 21, 23, 24, 28, 29, 31, 35, 37, 38]	12
Increase in readmission [32, 43]	2
Decrease in mortality [41]	1
No difference in mortality [34]	1
Decrease in error [22]	1
No difference in readmission [26]	1
Not reported	9
	27

## 4 Discussion

Our research identified six interventions (four themes and two observations): Standardized procedures (10/27, 37%) [23, 24, 29, 30, 31, 34, 35, 37, 39, 43], a decision tool for assessment of risk or discharge (7/27, 26%) [20, 26, 32, 36, 38, 40, 42], a checklist to assess risk of readmission (5/27, 19%) [19, 21, 28, 41, 44], the use of health information exchange (3/27, 11%) [18, 25, 27], meaningful use qualification [33] and electronic prescribing [22] each occurred 1/27, 4%. The standardized procedure showed the greatest level of benefit to quality of care, which was reported as an increase in communication, documentation, and improved clinical handoffs. Multiple decision tools, developed through Delphi teams, helped identify high‐risk factors for falls, discharge, and readmission. Finally, integrating health information exchange and electronic prescribing at the ED enabled shorter treatment times and higher patient satisfaction.

The findings of this review will inform the design and implementation of HIT tools to assist in quality improvement measures. Our findings are consistent with previous reviews: Clinical decision tools improved workflows and rates of compliance for imaging and medication orders. EHR tools save clinicians time, decrease duplicate testing, and result in some cost savings associated with duplicate tests [10, 11]. What is unique about the findings of this review are the improvements in communication, clinical data documentation, improved clinical handoffs, better prediction of readmission, and improved mortality.

Standardized procedures, protocols, checklists, HIE, and e‐prescribing integrated into the EHR improved quality of care without adversely affecting patient or staff satisfaction. Clinicians can leverage this information in the ED to improve quality of care. Administrators can look at these tools to save costs and gain efficiency. Policy makers can ensure regulation does not impede on the development and implementation of tools that improve quality.

The barriers to adoption listed are not insurmountable. Integration with the EHR simply requires the feature be listed by the vendor or internal EHR team for development. Feature development is a common procedure for software development. While cost seems daunting, leadership can overcome this. The organizational leadership can reprioritize funding for this important task. Finally, the last barrier listed was ED decision making. This only occurred in one article, and it related to the complexities of care beyond the ED. Due to this complexity, it is difficult to make decisions in a timely manner which could delay patient transfer. A solution to this barrier could involve decision trees and provider collaboration. Through collaborative efforts, providers could arrive at decisions more quickly.

One issue not addressed by the articles for analysis is the structural issues in each facility that addresses training and user acceptance of HIT. Such acceptance would include an overview of integration into workflow and operational habits. This structural issue could have large implications to the results of each study.

### 4.1 Limitations

Limitations of our findings are predicted due to heterogeneity in the articles studied and differences in the context in which HIT utilization was observed. A primary limitation of this review methodology is that a synthesis of findings across all articles is not entirely possible due to an inconsistency of reporting on effect sizes. Only two articles reported effect sizes, which made it impossible to conduct a meta‐analysis. Ideally, our review would have examined RCTs and other true experiments that were conducted in ED environments with clear results between interventions. However, these studies were not readily available for analysis.

Additionally, artificial intelligence and blockchain have been replete in the literature over the last few years, but these topics did not surface in our literature review. While artificial intelligence was added as a MeSH term in 1986, blockchain was not added until 2020. Future research should include these terms into their search string to ensure the results are exhaustive.

A subgroup analysis was not performed to determine how diverse groups of patients and providers would respond to different treatments and HIT integration. This would have been necessary at the study level to make a general determination. Because this was not performed at the study level, subgroup analysis was not possible at the systematic review level.

#### 4.1.1 Practical Factors in the Clinical Application of HIT

A limitation of this manuscript is based on differences between diverse types of emergency departments. Articles chosen to base the work upon were not specific regarding how their patient flow was managed, how staff was allocated, or any technical infrastructure of the departments. Generally, many of the utilized articles did not elaborate on these areas; as such, these results were not available for analysis. Disease complexity was not elaborated on in this manuscript as it was not discussed in the bulk of articles that were reviewed. Practically speaking, we treat the diseases that present themselves in any emergency department. Some of the articles chosen for the review were specific to a type of malady, but this was not the article's focus. A focus on specific diseases and the application of HIT to them is outside the scope of this study. Thus, the authors did not explore the impact of clinical context differences on implementing HIT measures. Again, this is outside the scope of this singular article. Such an examination would be left as a focus for singular articles focusing on a specific disease process and HIT, which we did not include in search terms. Our focus here was from a generalist perspective. Considering intervention measures from a medical staff perspective, this manuscript chose a generalist perspective. Unquestionably, it could be found in the literature that many issues exist in implementation, acceptance, training, operational habits, and other factors surrounding intervention effects. However, this was not the focus of this article. Any one of these areas would be a focus for a singular article on its own. It is well known that medical staff undergo training upon entering any workplace. Any concerns surrounding this training, or its implementation, are outside the scope of this manuscript. Intervention measures from a patient perspective was not a search term the authors included and thus was not a consideration in this manuscript. A patient‐focused manuscript looking at intervention measures and acceptance levels is well outside the scope of this study, which was more practitioner focused.

### 4.2 Future Research

Our findings suggest that there are significant benefits to the implementation and utilization of HIT on quality‐of‐care delivery, however implementation may be stunted by the complexity of integrating these tools into the EHR system. Further research is needed to assess aspects of health information that are not deemed user‐friendly to develop a more accessible system that may be adopted universally.

## 5 Conclusion

HIT implementation into emergency departments can improve quality of care and communication through standardized procedures, checklists, and decision tools, reduce the risk of medical error, and improve time to treatment without a decrease in either patient or staff satisfaction. Our review found that HIT can be useful in reviewing readmission metrics and enhancing communication between providers. This review found that HIT can have the greatest impact in documentation while improving individualized care for patients.

## Protocol and Registration

This review was registered with PROSPERO on July 31, 2020. The registration number is CRD42020201834. The review was conducted in accordance with the Kruse Protocol published in 2019 [14] and reported in accordance with PRISMA [45]. The AMSTAR 2 checklist was used to validate the review was not biased. This was provided as an attachment to the publisher.

## Author Contributions

**Clemens Scott Kruse:** conceptualization, investigation, funding acquisition, writing – original draft, writing – review and editing, visualization, validation, methodology, software, formal analysis, project administration, data curation, supervision, resources. **Michael Mileski:** conceptualization, methodology, validation, investigation, writing – review and editing. **Brooke Herzog:** conceptualization, methodology, writing – original draft. **Leah M. Frye:** conceptualization, methodology, writing – original draft. **Jackson R. Spencer:** conceptualization, methodology, writing – original draft. **Elizabeth A. Stevenson:** conceptualization, methodology, writing – original draft.

## Ethics Statement

This study does not fulfil the definition of human subjects' research and is therefore not subject to IRB oversight, in accordance with 45 CFR § 46.102.

## Conflicts of Interest

The authors declare no conflicts of interest.

## Transparency Statement

The lead author Clemens Scott Kruse affirms that this manuscript is an honest, accurate, and transparent account of the study being reported; that no important aspects of the study have been omitted; and that any discrepancies from the study as planned (and, if relevant, registered) have been explained.

## Supporting information

Appendix A.

Appendix B.

Appendix C.

Appendix D.

## Data Availability

The data that support the findings of this study are openly available in ‘The effect of health information technology on quality in the emergency department’ on Texas Dataverse at https://doi.org/10.18738/T8/OILH8H.
