# Supplementary material for: The Effects of Health Information Technology on Quality of Care in Emergency Departments: A Systematic Review
Source: Health Sci Rep. 2025 Jul 7;8(7):e70962. doi: 10.1002/hsr2.70962 (PMC12230508; doi:10.1002/hsr2.70962)
Supplement: Supplementary file 1 — Appendix A. [file HSR2-8-e70962-s001.docx]

Appendix A: Translation of observations of intervention, results, and medical outcomes to themes

| Authors | Intervention | **Intervention themes** | Results (compared to control group) | **Results themes** | Medical Outcomes Reported | **Medical outcomes themes** |
| --- | --- | --- | --- | --- | --- | --- |
| Bahous, et al.^18^ | The Health information Exchange (HIE) system; The referral letters; The patients’ medical history were analyzed to assess information gaps | Health information exchange | No control group. The following were indicators of key areas: The HIE system (x̄=54.1%); The referral letter (x̄=43.9%); The patient’s medical history (x̄=73.5%). | Decrease in time to treatment | The HIE, referral letters, and medical history can narrow information gaps and may reduce the time required for collecting patient’s medical information at time-critical points | Improve process of care |
| Byczkowski, et al.^19^ | None. Focus group identified through the EHR, and described through a survey dimensions of family-centered care important to parents in pediatric emergency care | Checklist | Researchers compared their results to published study populations. Family-centered care divided into eight dimensions: 1) emotional support, 2) coordination of care, 3) involve the family and patient in care decisions by prompting for and respecting care preferences, 4) alternative and timely care, 5) education, communication, and information, 6) management of pain, 7) child-focused and safe care, 8) continuity | Improved communication or reporting | Not reported. | Not reported |
| Chan, et al.^20^ | None. Decision tool developed using the EHR. | Decision tool | The control group was non readmissions compared based on patient demographics, mode of arrival, acuity of patient, status of category, seniority ranking of physician in charge, and medical diagnosis. Risk factors identified with returning within 72 hrs: older males who arrive by ambulance and triaged as P2, and diagnoses of heart problems, abdominal pain or viral infection (*p* < .001); Chinese ethnicity (*p* = .006). Seniority of physician showed no significant differences between groups (*p* = 0.419). | No improvement in readmission rates | Not reported. | Not reported |
| Horner, et al.^21^ | None. Checklist developed using EHR to predict the likelihood of imaging. | Checklist | No control group. Imaging was performed 29.7% of the visits; Isolated CT 13.4% of visits. There were differences by several factors: The Black (OR: 0.4 (95%CI: 0.4, 0.5)), Medicaid (OR: 0.6 (95%CI: 0.5, 0.7)) patients showed lower odds of advanced imaging when compared to white patients with private insurance, respectively. | No improvement in readmission rates | Racial and insurance disparities were present. General EDs are less likely than pediatric EDs to use advanced imaging overall. | Improve process of care |
| Khalil, et al.^22^ | The electronic prescribing and medication administration (MAR) reconciliation. | Electronic prescribing | Control group (n=54). Decreased average error rate from 4·41 to 0·52 err/patient (*p* < .0001) and .43-.05 errors per order (*p* < .005). | Decrease in error | Reduction in the severity of the errors. | Decrease in error |
| Manias, et al.^23^ | Transcribed telephone handoffs followed by EHR transcription. | Standardized procedure | No control group. Patient conditions do not deteriorate as quicky when effective telephone handoffs occurs during in-flight transfers followed by EHR transcription. | Improved communication or reporting | Deterioration of condition was reported in three calls | Improve process of care |
| Matthaeus-Kraemer, et al.^24^ | None. Standardized procedure developed for sepsis guidelines implementation (EHR provided records for group discussions) | Standardized procedure | No control group. The root cause of delayed detection was communication errors and handover difficulties throughout | Improved communication or reporting | Improved medical outcomes are associated with early recognition of sepsis followed by prompt therapy. Length of stay or mortality benefits not associated with 24/7 intensivist staffing | Improve process of care |
| Melvin, et al.^25^ | None. Survey instrument tested usability perceptions of the CeHa-HIE.. | Health information exchange | No control group. Most agreed the HIE was easy to use and its use added value to their work in the ED. Physicians asked for improved information integration into system EHR. | Positive perception of care or tool | Not reported. | Not reported |
| Tsai, et al.^26^ | None. Decision tool developed with the EHR. | Decision tool | No control group. | No difference in quality of care or readmission rates | Most patients visited the ED in the evening (51%); Identified as Triage Level III (76.5%) in their first ED visit. Causes of revisits followed by ICU admission were doctor-related (21/51, 41.1%), illness-related (18/51, 35.3%), or patient-related (12/51, 23.5%). Disease categories: neurological (23.5%), digestive (23.5%), | No difference in readmission |
| Everson, et al.^27^ | HIE. Researchers used the audit data from an EHR to capture the time span between the request for outside information and access of the data. Researchers assessed whether length of stay, odds of imaging, hospitalization, and total charge was mediated by the measured request-to-access time. | Health information exchange | No control group. Researchers found no direct association between mode of return of HIE information and ED outcomes. Use of the HIE was associated with faster request-to-access (x̄=58.5 minutes); and faster access resulted in faster ED service; Each 1 hour shorter access to information resulted in shorter ED stay (x̄=52.9 minutes), likelihood of imaging (x̄=2.5%, 1.6%, and 2.4% for CT, MRI, and radiographs, respectively), and likelihood of admission was 2.4% lower, (*p*<.001). | Decrease in time to treatment | Use of the HIE reduced time-to-access, which improved both care processes and ED utilization. | Improve process of care |
| Harris, et al.^28^ | Checklist for Parkinson's Disease and falls associated with other patients in ED. (EHR provided data) | Checklist | No control group. No statistically significant difference in triage code or representation rate between patients. Time to be seen x̅=23 min (SD 26); Time to be seen between business hours and after hours was x̅=20 (SD 21) and x̅=30 (SD 32), respectively. Patients under 80 yrs (n = 132; 51.0%) were admitted to hospital more frequently than those over 80 yrs (Pearson’s *X*2 test 162.2; df 1; p = 0.001). | Decrease in time to treatment | Documentation of medication regimes, adverse outcomes, and emergency care are improved when providers recognize a PD presentation. | Improve process of care |
| Josephy, et al.^29^ | Standardized procedure of single vs two physician sedation (EHR provided the pre data in the pre-post design) | Standardized procedure | Single physician sedation resulted in zero adverse events when compared with two physician team. | No difference in quality of care or readmission rates | Single physician sedation resulted in zero adverse events | Improve process of care |
| Newcomb, et al.^30^ | Prescribing protocol developed. User responses were compared on patient satisfaction surveys, specifically for pain complaints. (EHR provided patient matches) | Standardized procedure | No control group. The frequent ED users were 75% less likely to return a satisfaction survey than the infrequent ED users (*OR* = 0.2488; *p* < .0001). | Affected pts less likely to report satisfaction | ED providers can feel confident that they can withhold opioids without concern of adversely affecting patient satisfaction. | Improve process of care |
| Okafor, et al.^31^ | Standardized care transition process and EMR monitoring. | Standardized procedure | Pre intervention revealed 94 relevant missed clinical terms (MCI) while post intervention showed only 36 MCI with a similar number of care transitions. This was a 58% decrease in MCI without an increase in time for transitions. Users of the standardized procedure reported high satisfaction with care transitions. | Improved communication or reporting | The primary outcome measure was the number of missed clinical items. Both residents and faculty felt the intervention decreased the number of MCI. | Improve process of care |
| Bennet, et al.^32^ | Decision tool. The EHR provided the data. | Decision tool | Through a logistic regression, authors showed a statistically significant difference in 30-day readmission rates between groups. Rare conditions contributed significantly. | Decrease in readmission rates | Rare conditions resulted in more inpatient visits. | Increase in readmission |
| Brice, et al.^33^ | Meaningful Use of technology (MU). | Meaningful Use | The average EHR adoption among physicians was 43%. Post-acute utilization indicators improved. | No improvement in readmission rates | Not reported. | Not reported |
| Brauer, et al.^34^ | LACE model. The EHR provided data to the State Inpatient Database of the Agency for Healthcare Research and Quality (AHRQ), and Healthcare Cost and Utilization Project (HCUP) for Florida, California, and New York for the years 2006-2014, 2006-2011, and 2006-2013, respectively. | Standardized procedure | The rate of death or 30-day readmission was 14% (*p*<.494). The LACE model was a poor fit between groups (*C*=.631), and adding additional variables only slightly improved it. | No difference in quality of care or readmission rates | The rate of death or readmission within 30 days after discharge was not different between the groups (*C*=.631). | No difference in mortality |
| Bui, et al.^35^ | Standardized procedure (Barriers to Care). Two intervention exposures were compared (# contacts with case manager or community health worker: < 1/mo or >1/mo). | Standardized procedure | There was a higher systolic blood pressure in the high contact group. Medicaid participants in the high contact group had 42% (rate ratio (RR): 1.42; 95% CI: 1.08–1.86) and 64% (RR: 1.64; 95% CI: 1.08–2.48) greater risks for hospitalization and readmission than the low contact group, respectively. | No improvement in readmission rates | Medicaid participants in the high contact group had 42% (rate ratio (RR): 1.42; 95% CI: 1.08–1.86) and 64% (RR: 1.64; 95% CI: 1.08–2.48) greater risks for hospitalization and readmission than the low contact group, respectively. | Improve process of care |
| Curtis, et al.^36^ | History, Identify Red flags, Assessment, Interventions, Diagnostics, Reassessment, Communication (HIRAID) tested with ED nurses. | Decision tool | The self-efficacy score did not change between groups (x̅ (SD): 8.79(1.12) vs 9.03 (0.85), *t* = 0.91, *p* = .365). | No difference in quality of care or readmission rates | Not reported. | Not reported |
| Economos, et al.^37^ | Standardized procedure. The EHR provided data. Participants were categorized by actively dying or not-actively dying, based on provider comments. | Standardized procedure | There was no statistical difference between end-of-life care for actively and non-actively dying patients. | No improvement in readmission rates | Actively dying patients mostly suffered from vascular conditions (29.4%), were more likely to have decisions to withhold or withdraw treatments (*OR* = 5.3 [1.56; 20.7], *p* = .003), to have strong opioids (*OR* = 5.32 [2.1; 13.9], *p* <.0001), hypnotics (*OR* = 2.6 [0.95; 8.39], *p* = .05), scopolamine (*OR* = 2.5 [1.1; 6.13], *p* = .03), were less likely to have unbeneficial treatments in terminal conditions, such as resuscitation care (*OR* = 0.06 [0.001; 0.52], *p* = .002) and antibiotics (*OR* = 0.42 [0.19; 0.92], *p* = .022). | Improve process of care |
| Martinez-Sanchez, et al.^38^ | Quality decision tool. Researchers evaluated eight quality indicators (QI) for care received prehospital ED. Data collected from the EHR. | Decision tool | Intervention teams: (EMT + ALSn, technician + ALSn, or technician + physician) Symptoms of poisoning were present in 124 patients (48.8%) and severe in 42 (16.5%). The treatments given were oxygen therapy (n=82; 32.3%), fluid therapy (n=46; 18.1%) and administration of antidotes (n=17; 6.7%). | Decrease in time to treatment | Decreased time to treatment. | Improve process of care |
| Olino, et al.^39^ | Standardized procedure. Transfer Notes (NT) in the EHR along with Modified Early Warning Scores (MEWS) analyzed for effective communication. | Standardized procedure | No control group. The NT was performed at 95% in only two months (Jan-Feb). The MEWS was recorded in 85.6% (n = 6,870) of the EHR records. The MEWS remained unchanged in 96.8% (n = 6,652) EHR records. | Improved communication or reporting | Not reported. | Not reported |
| Yakusheva, et al.^40^ | Decision tool from the READI (Readiness Evaluation And Discharge Interventions) study. Patient discharge data from the EHR provided eight item short form of READI. The 30-day readmission or ED visit was used as a categorical variable. | Decision tool | No control group. There was a negative association between nurse productivity and the likelihood of a readmission (−0.48 percentage points, *p*<.001) and an ED visit (−0.29 percentage points, *p*=.042). The variability in nurse productivity explained only 9.07% of variance in patient discharge readiness scores. | Negative productivity | Not reported. | Not reported |
| Delawder, et al.^41^ | QI Checklist -- sepsis prevention built into EHR. Develop and implement an interdisciplinary team to address early implementation of sepsis bundles in the emergency department and to compare sepsis bundle compliance 3 months pre- and 3 months postintervention implementation. | Checklist | The intervention showed an improvement in time to each bundle element, with the exception of antibiotics and blood cultures. Changes were also observed in meeting bundle compliance in fluid resuscitation volume (χ2 = 16.3, *p*≤.001): initial lactate collected within 180min (χ2 = 11.3,*p*≤ .01) and time to second lactate within 360min (χ2 = 27.7,*p*≤ .001). | Decrease in time to treatment | Mortality rates declined from 12% to 5%. | Decrease in mortality |
| Dimeff, et al.^42^ | VCAM decision tool integrated with EHR. The “Dr. Dave” avatar was an iterative, user-centered design. This along with other patient-facing tools, provider-facing tools, and a clinical decision support (CDSS) tool were tested to aid discharge disposition. | Decision tool | No control group. Patients scored highly on the Perception of Care domain (12/18, 66.7%) endorsed this domain at a medium to high level. The inter-rater reliability Kappa = 0.900; *p* < .0001). | Positive perception of care or tool | Not reported | Not reported |
| Munjal, et al.^43^ | Standardized procedure. Measured outcomes: EMS transported to home; 72 hr and 30-day ED revisits and 30-day readmissions. Data provided by the EHR. | Standardized procedure | A higher rate of 30-day ED revisit observed in the intervention group (18.52% vs. 10.49%; *OR* 1.939; *p* = .043). A higher rate of 72-hr ED returns was also observed by EMS-transported discharges (2.47% vs. 0.62%; *OR* 4.076; *p*=.21) and 30-day readmissions (12.35% vs. 6.17%; *OR* 2.141; *p*=.06). This result was not statistically significant. | No improvement in readmission rates | Discharges transported home by EMS are at a higher risk of 30-day ED revisit, 72 hr ED return, and 30-day readmission. | Increase in readmission |
| Wooldridge, et al.^44^ | Systems Engineering Initiative for Patient Safety (SEIPS) checklist. It was developed using semi-structured interviews. | Checklist | The checklist development process identified nine dimensions of work system barriers and facilitators in care transitions of pediatric trauma patients from the ED to the operating room (13 cases), operating room to PICU (12 cases) and ED to PICU (9 cases): ED decision making, anticipation, role ambiguity, interacting with family, staffing/resources, physical environment, technology, team cognition, and characteristic of trauma care. At least one dimension was observed in all work-system elements. | Improved communication or reporting | Not reported. | Not reported |
