# Supplementary material for: The Effects of Health Information Technology on Quality of Care in Emergency Departments: A Systematic Review
Source: Health Sci Rep. 2025 Jul 7;8(7):e70962. doi: 10.1002/hsr2.70962 (PMC12230508; doi:10.1002/hsr2.70962)
Supplement: Supplementary file 2 — Appendix B. [file HSR2-8-e70962-s003.docx]

Appendix B: Translation of observations of patient/staff satisfaction, quality outcomes, and barriers to adoption to themes

| Authors | Patient or Staff Satisfaction | **Satisfaction themes** | Indicators of quality | **Quality themes** | Barriers to adoption | **Barrier themes** |
| --- | --- | --- | --- | --- | --- | --- |
| Bahous, et al.^18^ | Not reported | Not reported | Multiple sources of information fill in missing clinical data from the others, and the EHR makes these available upon demand. No one data source is comprehensive. | Increase in communication or documentation | Must have HIE integrated with EHR | Requires integration with EHR |
| Byczkowski, et al.^19^ | Positive effect on patient experience | Satisfied or highly satisfied | Not reported | Not reported | Not reported | Not reported |
| Chan, et al.^20^ | Not reported | Not reported | Readmission associated with age, gender, race, but not with physician or interface with HIT | Decrease in ED utilization | Not reported | Not reported |
| Horner, et al.^21^ | Not reported | Not reported | Racial and insurance disparities were present. General EDs are less likely than pediatric EDs to use advanced imaging overall | Increase in communication or documentation | Not reported | Not reported |
| Khalil, et al.^22^ | The majority of the respondents were satisfied with new service and felt it had resulted in faster and accurate charting of inpatient medications. Pharmacists can save more than 30 min per patient for the admitting officers. | Satisfied or highly satisfied | The average error rate decreased from 4·41 to 0·52 errors per patient (P < 0·0001) and 0·43-0·05 fewer errors per order (P < 0·005). | Decrease in error | Not reported | Not reported |
| Manias, et al.^23^ | Not reported | Not reported | Crutial information was missing from calls, no standardization of communication handoff | Increase in communication or documentation | Not reported | Not reported |
| Matthaeus-Kraemer, et al.^24^ | Not reported | Not reported | Intra- and interunit handovers in both prehospital and hospital care could positively impact the early detection and treatment of severe sepsis and septic shock. | Increase in communication or documentation | Integration with EHR |  |
| Melvin, et al.^25^ | Generally satisfied | Satisfied or highly satisfied | Ease of use, value of time | Ease of use | Integration of HIE into EHR | Requires integration with EHR |
| Tsai, et al.^26^ | Not reported | Not reported | The causes of revisits with ICU admission were judged to be doctor-related (21/51, 41.1%), illness-related (18/51, 35.3%), or patient-related (12/51, 23.5%) | Not associated with readmission | Not reported | Not reported |
| Everson, et al.^27^ | Not reported | Not reported | Reduction in ED care. For each 1-hour reduction in access (to data) time, visit length was decreased by 52.9 minutes, the likelihood of imaging was lower (by 2.5), and the likelihood of admission was 2.4% lower, average charges were $1,187 lower (*P* < .001) | Efficiency | EHR must have HIE capability | Requires integration with EHR |
| Harris, et al.^28^ | Not reported | Not reported | Improved detection, management and a multidisciplinary approach for people with PD along with strict medication regime adherence is likely to improve safety, quality of life, reduce symptom aggravation and ongoing risk of falls. | Increase in communication or documentation | Not reported | Not reported |
| Josephy, et al.^29^ | Not reported | Not reported | Single physician sedation resulted in zero adverse events | Increase in communication or documentation | Not reported | Not reported |
| Newcomb, et al.^30^ | Patient satisfaction with nursing care in the ED is dependent on wait time, precautions to protect safety, ability of staff to convey caring, compassion of caregivers. Patient satisfaction with providers is more dependent on ED cleanliness, pain control, wait time, and satisfaction with nurses. | Satisfied or highly satisfied | Maintaining high patient satisfaction is possible with communication, compassion, and cleanliness. | Increase in communication or documentation | Not reported | Not reported |
| Okafor, et al.^31^ | Patients reported a positive or neutral effect on satisfaction with care transitions. | Positive perception of care | Mean missed clinical items decreased by 58% without lengthening checkout duration. | Increase in communication or documentation | Not reported | Not reported |
| Bennet, et al.^32^ | Not reported | Not reported | Condition status contributed significantly to readmission rates | Decrease in readmission | Not reported | Not reported |
| Brice, et al.^33^ | Not reported | Not reported | MU was not associated with a decrease in readmission | Not associated with readmission | Cost, organizational disruption | Cost |
| Brauer, et al.^34^ | Not reported | Not reported | No difference in mortality | No difference in mortality | Does not integrate into EHR | Requires integration with EHR |
| Bui, et al.^35^ | Not reported | Not reported | participants in the high contact group were younger, were more likely to be female, black race, and current smokers and had higher diastolic blood pressure, BMI, and HbA1c level. In the analyses of the contact intensity and healthcare utilization (Fig. 1) adjusted for age, sex, race, baseline ACG risk score, and baseline healthcare utilization rate, Medicaid participants in the high contact group had 42% (rate ratio (RR): 1.42; 95% CI: 1.08–1.86) and 64% (RR: 1.64; 95% CI: 1.08–2.48) higher risks for hospital admission and readmission, respectively, than the low contact group. | Increase in communication or documentation | Not reported | Not reported |
| Curtis, et al.^36^ | Emergency personnel were highly satisfied with the HIRAID framework | Satisfied or highly satisfied | Improved clinical documentation, appropriate reassessments, improved monitoring, improved clinical handover | Increase in communication or documentation | Integration with EHR |  |
| Economos, et al.^37^ | Not reported | Not reported | End-of-life checklists / templates might improve hydration management, drug administration routes, and broncho-pulmonary secretions management | Decrease in error | Integration of templates into EHR and training staff |  |
| Martinez-Sanchez, et al.^38^ | Not reported | Not reported | Full teams (ALSp or ALSn + RIV) are more effective at treating poison in adolescents in 3 of 8 QI categories | Increase in communication or documentation | Integrating teams into procedures, cost of full teams | Cost |
| Olino, et al.^39^ | Not reported | Not reported | TNs fell slightly below target in 10 of the 12 months analyzed (80% compliance). MEWS performed 85.6% of cases. Of these patients, 96.8% had unchanged MEWS. No effect on safety. | Increase in communication or documentation | Busy schedules and ED overcrowding complicate creation of these important quality / safety tools. | Requires integration with EHR |
| Yakusheva, et al.^40^ | Not reported | Not reported | Low productivity nurses were the least efficient discharging patients through the ED. Medium productivity nurses demonstrated a 2.44 app reduction in ED visits without a readmission. High productivity nurses had a .86 app reduction in readmission and nonsignificant change in ED visits without readmission. | Efficiency | Not reported | Not reported |
| Delawder, et al.^41^ | Not reported | Not reported | Decrease in mortality from 12% - 5%. | Decrease in mortality | Not reported | Not reported |
| Dimeff, et al.^42^ | Patients expressed positive perception of care. Providers were very pleased with system. medical providers viewed the system as an efficient, effective, and safe method of improving care for suicidal ED patients and reducing unnecessary hospitalization. | Positive perception of care | Providers found the system to be efficient and effective, and safe. Reduced readmission, improved mortality. | Efficiency | Requires additional integration into EHR. | Requires integration with EHR |
| Munjal, et al.^43^ | Not reported | Not reported | Risk of readmission is higher for those transported home by EMS. | Not associated with readmission | Cost of EMS transport is high | Cost |
| Wooldridge, et al.^44^ | Not reported | Not reported | Not reported | Not reported | Anticipation, ED decision making, Interacting with family, Physical environment, Role ambiguity, Staffing resources, Team cognition, Technology, Characteristics of trauma care | Affects ED decision making |
