# Supplementary material for: The Effects of Health Information Technology on Quality of Care in Emergency Departments: A Systematic Review
Source: Health Sci Rep. 2025 Jul 7;8(7):e70962. doi: 10.1002/hsr2.70962 (PMC12230508; doi:10.1002/hsr2.70962)
Supplement: Supplementary file 3 — Appendix C. [file HSR2-8-e70962-s002.docx]

Appendix C: Other data collected on articles

| Authors | Sample Size | Bias within study | Effect Size | Country of Origin | Statistics Used | Strength of Evidence | Quality of Evidence | Source of Funding |
| --- | --- | --- | --- | --- | --- | --- | --- | --- |
| Bahous, et al.^18^ | 170 | Not observed | Not reported | Israel | Cronbach's alpha | III | B | Not reported |
| Byczkowski, et al.^19^ | 68 | One location in one country | Not reported | United States | Language processing, descriptive statistics | II | B | Agency for Healthcare Research and Quality |
| Chan, et al.^20^ | 104,751 | Singapore only | Not reported | Singapore | Multivariate analysis and GLM | III | A | Unfunded |
| Horner, et al.^21^ | 21,152 | One location in one country | Odds Ratio for individual variables | United States | multivariable logistic regression | III | B | National Institutes of Health |
| Khalil, et al.^22^ | 55 | Not observed | Not reported | Australia | Fisher's exact test | III | B | Victorian Health and Human Services |
| Manias, et al.^23^ | 127 | One location in one country | Not reported | Australia | Language processing, descriptive statistics | III | B | Australian Research Council |
| Matthaeus-Kraemer, et al.^24^ | 29 | Five multicenter focus groups in one country | Not reported | Germany | Language processing, descriptive statistics | III | B | German Federal Ministry of Education Research |
| Melvin, et al.^25^ | 48 | Surveyed only users of one HIE | Not reported | United States | Language processing, descriptive statistics | III | B | National Institutes of Health |
| Tsai, et al.^26^ | 51 | One ED in one country | Not reported | Taiwan | Descriptive statistics | III | B | Not reported |
| Everson, et al.^27^ | 2163 | Not reported | Not reported | United States | Multivariate analysis | III | A | Agency for Healthcare Research and Quality |
| Harris, et al.^28^ | 129 | One metropolitan 300 bed district hospital only | Not reported | Australia | Descriptive statistics | II | B | Unfunded |
| Josephy, et al.^29^ | 381 | Single facility | Not reported | United States | Descriptive statistics | III | B | Unfunded |
| Newcomb, et al.^30^ | 610 | Not reported | Not reported | United States | Descriptive statistics | III | A | Not reported |
| Okafor, et al.^31^ | 448 | Potential Hawthorne effect from patients | Not reported | United States | Descriptive statistics | I | A | Not reported |
| Bennet, et al.^32^ | 5389 (study) and 10,788 (matched cohort) | One location in one country | Not reported | United States | Logistic regression, and descriptive statistics | II | A | Not reported |
| Brice, et al.^33^ | 160 Hospitals | US only | Not reported | United States | Multilevel model (MLM), Generalized linear mixed models (GLMM), and descriptive statistics | II | A | Institute on  Healthcare Systems of the Schneider Institutes for Health Policy at Brandeis  University |
| Brauer, et al.^34^ | 440,742 | One location in one country | Not reported | United States | Multivariate logistical regression | I | A | National Cancer Institute |
| Bui, et al.^35^ | 1,342 | One facility, one location, one region of a country | Not reported | United States | Descriptive statistics | II | A | Center for Medicare and Medicaid Services |
| Curtis, et al.^36^ | 136 emergency nurses and 54 medical officers (190) | One location in one country | Cohen’s *d* = 0.5; medium | Australia | Descriptive statistics | II | A | Agency for Clinical Innovation |
| Economos, et al.^37^ | 146 | Not reported | Not reported | France | Odds ratios | III | B | Unfunded |
| Martinez-Sanchez, et al.^38^ | 254 | care report summaries of the SEM involve a loss of information | Not reported | Spain | Descriptive statistics, confidence intervals | III | B | Not reported |
| Olino, et al.^39^ | 8,021 | Not reported | Not reported | Brazil | Descriptive statistics | III | A | Not reported |
| Yakusheva, et al.^40^ | 522 Nurses, 29,986 patients | Nurses were not randomly assigned to patient care, limiting causal inference. Also do not have information on all nurses and other providers who may have contributed to a patient’s discharge readiness. Lastly, although the study used a multisite geographically diverse non disease/condition-specific patient sample, generalizability is limited due to selection of Magnet hospitals. | Cohen’s *f*^2^ = .02; small | Saudi Arabia | 2-tailed significance test | III | A | Not reported |
| Delawder, et al.^41^ | 214 | Not reported | Not reported | United States | Chi Square | III | B | Not reported |
| Dimeff, et al.^42^ | 21 | Not reported | Not reported | United States | Qualitative research methodology | III | B | Not reported |
| Munjal, et al.^43^ | 207 | Limited to one urban medical center in one region of the US. Reasons for EMS transport unknown. Being chosen for transport home by EMS could be the same risk factors that cause the higher risk of readmission. | Not reported | United States | Descriptive statistics | III | A | National  Heart, Lung, & Blood Institute |
| Wooldridge, et al.^44^ | 34 | One region of one country | Not reported | United States | Language processing, descriptive statistics | III | B | Agency for Healthcare Research Quality |
