# Supplementary material for: The Effects of Health Information Technology on Quality of Care in Emergency Departments: A Systematic Review
Source: Health Sci Rep. 2025 Jul 7;8(7):e70962. doi: 10.1002/hsr2.70962 (PMC12230508; doi:10.1002/hsr2.70962)
Supplement: Supplementary file 4 — Appendix D. [file HSR2-8-e70962-s004.docx]

Appendix D: Rejected articles with reasons for rejection

| Date of pub | Authors | Study title | Journal | Reason for Rejection |
| --- | --- | --- | --- | --- |
| 2020 | Mullins, Alexandra; O'Donnell, Renee; Mousa, Mariam; Rankin, David; Ben-Meir, Michael; Boyd-Skinner, Christopher; Skouteris, Helen | Health Outcomes and Healthcare Efficiencies Associated with the Use of Electronic Health Records in Hospital Emergency Departments: a Systematic Review. | Journal of Medical Systems | Review |
| 2019 | Judkins, Simon | Clear guidance on improving care in Emergency Departments: Clinical pharmacists as part of the team. | Journal of Pharmacy Practice & Research | No Outcomes Reported |
| 2018 | Elder, Joshua W.; DePalma, Garrett; Pines, Jesse M. | Optimal Implementation of Prescription Drug Monitoring Programs in the Emergency Department. | Western Journal of Emergency Medicine: Integrating Emergency Care with Population Health | Review |
| 2016 | Uspal, Neil G.; Rutman, Lori E.; Kodish, Ian; Moore, Ann; Migita, Russell T.; Walthall, Jennifer D. H. | Use of a Dedicated, Non-Physician-led Mental Health Team to Reduce Pediatric Emergency Department Lengths of Stay. | Academic Emergency Medicine | Not Germane to Objective |
| 2019 | Rizk, Elsie; Swan, Joshua T; Fink, Ezekiel | Prioritization of quality indicators for opioid stewardship | American Journal of Health-System Pharmacy | No Outcomes Reported |
| 2015 | Jennings, Natasha; Gardner, Glenn; O'Reilly, Gerard; Mitra, Biswadev | Emergency NP Model of Care in an Australian Emergency Department. | Journal for Nurse Practitioners | Not Germane to Objective |
| 2020 | O'Reilly GM, Mitchell RD, Noonan MP, Hiller R, Mitra B, Brichko L, Luckhoff C, Paton A, Smit V, Santamaria MJ, Cameron PA. | Informing emergency care for COVID-19 patients: The COVID-19 Emergency Department Quality Improvement Project protocol | Emerg Med Australas | Protocol |
| 2020 | Tan A, Durbin M, Chung FR, Rubin AL, Cuthel AM, McQuilkin JA, Modrek AS, Jamin C, Gavin N, Mann D, Swartz JL, Austrian JS, Testa PA, Hill JD, Grudzen CR; Group Authorship: Corita R. Grudzen on behalf of the PRIM-ER Clinical Informatics Advisory Board. | Design and implementation of a clinical decision support tool for primary palliative Care for Emergency Medicine (PRIM-ER) | BMC Med Inform Decis Mak | Not Germane to Objective |
| 2020 | Horberg MA, Certa JM, Rubenstein KB, Hurley LB, Satre DD, Kadlecik PM, Silverberg MJ. | Beyond the HIV Care Continuum and Viral Suppression: Broadening the Scope of Quality Metrics for Total HIV Patient Care | AIDS Patient Care STDS | Not Germane to Objective |
| 2020 | Rosenkrantz AB, Smith SW, Recht MP, Horwitz LI. | Perceptions of Radiologists and Emergency Medicine Providers Regarding the Quality, Value, and Challenges of Outside Image Sharing in the Emergency Department Setting | AJR Am J Roentgenol | Not Germane to Objective |
| 2016 | Kern LM, Edwards A, Kaushal R. | The Patient-Centered Medical Home and Associations With Health Care Quality and Utilization: A 5-Year Cohort Study | Ann Intern Med | Not Germane to Objective |
| 2018 | Marcin JP, Romano PS, Dharmar M, Chamberlain JM, Dudley N, Macias CG, Nigrovic LE, Powell EC, Rogers AJ, Sonnett M, Tzimenatos L, Alpern ER, Andrews-Dickert R, Borgialli DA, Sidney E, Casper C, Dean JM, Kuppermann N; Pediatric Emergency Care Applied Research Network. | Implicit Review Instrument to Evaluate Quality of Care Delivered by Physicians to Children in Emergency Departments | Health Serv Res | Not Germane to Objective |
| 2018 | Mundt MP, Zakletskaia LI. | Putting the pieces together: EHR communication and diabetes patient outcomes | Am J Manag Care | Not Germane to Objective |
| 2016 | Padrez KA, Ungar L, Schwartz HA, Smith RJ, Hill S, Antanavicius T, Brown DM, Crutchley P, Asch DA, Merchant RM. | Linking social media and medical record data: a study of adults presenting to an academic, urban emergency department | BMJ Qual Saf | Not Germane to Objective |
| 2018 | Kraaijvanger N, Rijpsma D, Roovers L, van Leeuwen H, Kaasjager K, van den Brand L, Horstink L, Edwards M. | Development and validation of an admission prediction tool for emergency departments in the Netherlands | Emerg Med J | Model (not a study) |
| 2018 | Colicchio TK, Fiol GD, Stoddard GJ, Narus SP. | Evaluation of a systematic methodology to detect in near real-time performance changes during electronic health record system implementations: a longitudinal study | AMIA Annu Symp Proc | Not Germane to Objective |
| 2017 | Balestra | Electronic Health Records: Patient Care and Ethical and Legal Implications for Nurse Practitioners | J for Nurse Pract | Not Germane to Objective |
| 2018 | Judson T, Press M, Detsky A | Saving without compromising: Teaching trainees to safely provide high value care | Healthcare | Not Germane to Objective |
| 2019 | Cameron P, Fitzgerald M, Zalstein S | Over view of major traumatic injury in Australia––Implications for trauma system design | Injury | Not Germane to Objective |
| 2020 | Kleinpell R, Kapu A, Wo Y | The use of national collaborative to promote advanced practice registered nurse-led high-value care initiatives | Nursing Outlook | Not Germane to Objective |
| 2019 | Holloway-Kew K, Baker T, Pasco J | The epidemiology of emergency presentations for falls from height across Western Victoria, Australia | Australasian Emer Care | Not Germane to Objective |
| 2018 | Hamilton J, Heads A | Ethnic differences in the diagnosis of schizophrenia and mood disorders during admission to an academic safety-net psychiatric hospital | Psychiatry Res | Not Germane to Objective |
| 2019 | Geisler B, Pietzsch J | Model to evaluate the impact of hospital-based interventions targeting false-positive blood cultures on economic and clinical outcomes | J of Hosp Infection | Model (not a study) |
| 2017 | Perron C, Bachur R, Stack A | Development, Implementation, and Use of an Emergency Physician Performance Dashboard | Clinical Ped Em Med | Not Germane to Objective |
| 2020 | Willemse S, Smeets W | Spiritual care in the intensive care unit: An integrative literature research | J of Crit Care | Not Germane to Objective |
| 2020 | Du G, Zhang J, Li S | Joint imbalanced classification and feature selection for hospital readmissions | Knowledge-Based systems | Conference proceding |
| 2016 | McHugh M | The Impact of the Affordable Care Act on Health Care Alliances’ Quality Improvement Efforts in Targeted Communities: Perceptions of Health Care Alliance Leaders | The Joint Commission Journal on Quality and Pt Safety | Not Germane to Objective |
| 2021 | Ayalew B, | The Health of Children in Immigrant Families: Key Drivers and Research Gaps through an Equity Lens | Academic Pediatrics | Not Germane to Objective |
| 2016 | Simon A | Asthma Action Plan Receipt among Children with Asthma 2-17 Years of Age, United States, 2002-2013 | The J of Pediatrics | Not Germane to Objective |
| 2017 | Edaibat E | System dynamics simulation modeling of health information exchange (HIE) adoption and policy intervention: A case study in the State of Maryland | Operations Research for HC | Not Germane to Objective |
| 2018 | Soltis_jarrett V | The TANDEM3-PC: The Foundation for an Innovative, Integrated Behavioral Health NP-led Model of Practice in Rural Primary Care | Arch of Psychatric Nurs | Model (not a study) |
| 2017 | Narayanamurthy G | Is the hospital lean? A mathematical model for assessing the implementation of lean thinking in healthcare institutions | Operations Research for HC | Model (not a study) |
| 2016 | Wooldridge A, Carayon P, Hoonakker P | SEIPS-based process modeling in primary care | Applied Erg | Model (not a study) |
| 2016 | Bussieres A | The Treatment of Neck Pain–Associated Disorders and Whiplash-Associated Disorders: A Clinical Practice Guideline | J of Manipulative and Phsysiological Therapies | Not Germane to Objective |
| 2019 | Gomez-Barrado J, Gomez-Turegano P | Communication between levels of care: An opportunity for improvement in cardiovascular prevention | Clinca e Investigacion en Arteriosclerosis | Not Germane to Objective |
| 2018 | Citron I, Saluja S, Steer M | Surgical quality indicators in low-resource settings: A new evidence-based tool | Surgery | Not Germane to Objective |
| 2018 | Bird Cloe, Manocchia M, Fremont A | Mapping the Gaps: Gender Differences in Preventive Cardiovascular Care among Managed Care Members in Four Metropolitan Areas | Women's Health Issues | Not Germane to Objective |
| 2018 | Daniel O | Effects of health information technology and health information exchanges on readmissions and length of stay | Health Policy and Tech | Not Germane to Objective |
| 2016 | Jenerette C, Mayer D | Patient-Provider Communication: the Rise of Patient Engagement | Seminars in Oncology Nurs | Not Germane to Objective |
| 2020 | Duncan G, Darzins P | Protocol for a randomised controlled trial evaluating the impact of a community pharmacy discharge medication reconciliation service on unplanned hospital readmissions – The DCMedsRec trial | Research in Social and Admin Pharm | Protocol |
